# Structural relaxation and domain formation in anisotropically strained La_0.7_Sr_0.3_MnO_3_/LaFeO_3_ superlattices on DyScO_3_(101)

**DOI:** 10.1038/s41598-026-35436-2

**Published:** 2026-01-13

**Authors:** Yu Liu, Thea Marie Dale, Emma van der Minne, Susanne Boucher, Romar Avila, Christoph Klewe, Gertjan Koster, Magnus Nord, Mari-Ann Einarsrud, Ingrid Hallsteinsen

**Affiliations:** 1 Department of Materials Science and Engineering, NTNU Norwegian University of Science and Technology, Trondheim, Norway; 2 Department of Physics, NTNU Norwegian University of Science and Technology, Trondheim, Norway; 3 MESA + Institute for Nanotechnology, University of Twente, Enschede, The Netherlands; 4 Advanced Light Source, Lawerence Berkeley National Laboratory, Berkeley, CA US

**Keywords:** Anisotropic strain engineering, Transition metal oxide thin film, Strain relaxation, Structural twin domain, Materials science, Physics

## Abstract

Anisotropic strain engineering in epitaxial oxide films provides new opportunities to control the antiferromagnetic and structural properties crucial for advancements of antiferromagnetic spintronics. Here we report on a (La_0.7_Sr_0.3_MnO_3_/LaFeO_3_)_4_ superlattice grown on (101)_o_ DyScO_3_ substrate which imposes significant anisotropic in-plane strain. Reciprocal space mapping reveals selective strain relaxation along the tensile in-plane [010]_o_ axis, while compression along the perpendicular in-plane [$\:\stackrel{-}{1}01$]_o_ axis remains strained. Scanning precession electron diffraction and higher-order Laue zone analysis show that the relaxation is accommodated by structural domain formation in the LaFeO_3_ layers, initiating from the second bilayer and propagating out-of-plane. These domains minimise structural defects and correlate with the substrate step edges. X-ray magnetic dichroism measurements reveal bulk-like in-plane antiferromagnetic order with polydomain signature as previously reported. Our findings reveal the presence of structural domains coexisting with antiferromagnetic polydomain states, showing a strain-domain-magnetism relationship that provides insights for applications of strain engineering in spintronics applications.

**Supplementary Information:**

The online version contains supplementary material available at 10.1038/s41598-026-35436-2.

## Introduction

Antiferromagnetic (AF) materials are rapidly emerging as key candidates for next-generation spintronic technologies^1,2^. Unlike ferromagnets, AF materials are spin-compensated with magnetic sublattice, giving no macroscopic magnetisation. This intrinsic spin compensation eliminating stray field and enabling ultrafast spin dynamics, offers major advantages for energy-efficient and high-speed devices. However, the absence of macroscopic magnetic moment makes the spin orientation difficult to control. In addition, the lattice imperfections and crystal twinning often results in AF multidomain formation. Thus, AF materials have primarily been used as passive components such as pinning layers for the active ferromagnets for exchange bias applications^3,4^.

Complex oxide perovskites, such as LaFeO_3_ (LFO) and La_0.7_Sr_0.3_MnO_3_ (LSMO), exhibit crystal structures composed of corner-sharing BO_6_ octahedra that are highly responsive to epitaxial strain. LFO is a G-type antiferromagnet in which strain-induced distortions modify the magnetocrystalline anisotropy and enable control over the Néel vector^5,6^. LSMO, a double-exchange ferromagnet, similarly exhibits strain-tunable magnetic anisotropy^7,8^, making both materials well suited for studying strain–spin coupling in perovskite thin films. By choosing a low-symmetry substrate, the AF domain degeneracy can be broken in epitaxial thin films^9^. For example, orthorhombic perovskite substrates such as DyScO_3_ (DSO) and GdScO_3_ impose anisotropic biaxial strain on LFO, where the strain is compressive in one axis, but tensile in the other^5^. LFO films strained on (101)-oriented DSO experiences + 2.94% tensile strain alng one in‐plane axis and − 0.22% compression alongthe perpendicular axis. This strain engineering results in lowering of the symmetry from orthorhombic to monoclinic and forces the structural and AF domain from usually polydomain configuration to a single domain.

The combination of different perovskites in thin film heterostructures, e.g., ferromagnetic LSMO and antiferromagnetic LFO, adds another opportunity to control the AF order parameter. X-ray absorption spectroscopy measurements reveal that near an LSMO/LFO interface, the Fe magnetic moments in LFO become canted, and produce a net interfacial magnetisation in the LFO layer^10^. This magnetic reconstruction is due to the oxygen-octahedral connectivity, where perovskite tilt patterns propagate across the heterointerface (so‐called octahedral coupling). In LSMO/LFO superlattices, octahedral coupling can alter the Mn–O–Mn and Fe–O–Fe bond angles, thereby changing the local magnetic anisotropy and the Curie temperature^11^. In this way, the interface in heterostructures provides a leverage to tune AF spin order by modifying structural distortions and exchange paths across thelayers.

In this study, we investigate the structural and magnetic properties of a (La_0.7_Sr_0.3_MnO_3_ /LaFeO_3_)_4_ superlattice grown on a (101)_o_ DSO substrate, which imposes highly anisotropic in-plane strain. A combination of high-resolution X-ray diffraction (XRD), reciprocal space mapping (RSM), scanning precession electron diffraction (SPED)^12^, and higher-order Laue zone (HOLZ) analysis were used to probe the strain relaxation mechanisms and structural domain formation^13^. Our findings reveal that the LFO layers undergo anisotropic strain relaxation, forming structural domains that propagate throughout the superlattice, while LSMO remains partially strained. Additionally, X-ray magnetic circular (XMCD) and linear dichroism (XMLD) measurements show bulk-like AF behaviour. By exploring the interplay between anisotropic strain and structural relaxation, this study contributes to a broader understanding of strain-driven effects in complex oxide superlattices.

## Results and discussion

### Strain analysis

**Fig. 1:**
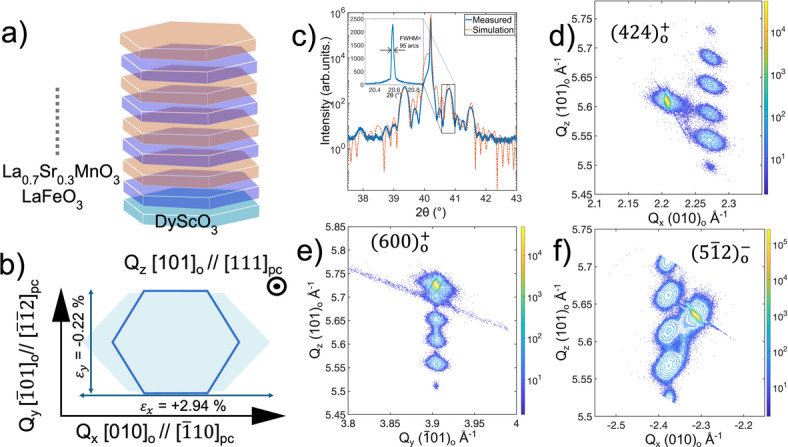
(**a**) Schematic of the superlattice with stacking order, with LFO in blue and LSMO in orange colour. (**b**) Illustration of the lattice mismatch in (101)_o_ orientation between DSO (light blue) and LFO (solid blue line). Q_x, y,z_ orientations are given in orthorhombic and pseuduocubic notations. (**c**) 2 theta-omega line scan and simulation of symmetrical (202)_o_ peak with inset showing rocking curve of the selected superlattice peak with FWHM = 95 arcseconds. (**d**-**f**) Reciprocal space maps of, respectively, $\:{\left(424\right)}_{\mathrm{o}}^{+}$, $\:{\left(600\right)}_{\mathrm{o}}^{+}$ and $\:(5\overline{1}2)_{o}^{-}$ reflections.

Superstructures of (LSMO/LFO)_4_ on DSO(101)_o_ substrates were grown by pulsed laser deposition (PLD) as schematically shown in Fig. 1a, with growth parameters optimised from previous works^5,7^. The strain state is illustrated in Fig. 1b for LFO on DSO substrate with a tensile strain of 2.94% in te [010]_o_ in-plane axis and a compressive strain of -0.22% in the $\:[\overline{1}01]_{\mathrm{o}}$ perpendicular in-plane axis. For LSMO on DSO there is tensile strain in both directions, respectively, 4.06% in te [010]_o_ in-plane axis and 0.81% in the $\:[\overline{1}01]_{\mathrm{o}}$ perpendicular in-plane axis.

Symmetrical X-ray diffraction (XRD) 2theta-omega line scans around the (202)_o_ peak of DSO shown in Fig. 1c reveals periodic superlattice peaks with Kiessig fringes in between, indicating a highly ordered crystalline thin film. The periodic lattice peak angles show a bilayer thickness of 13.5 nm and total superstructure thickness of 54 nm. The simulated superlattice line scan (Fig. 1c) using InteractiveXRDFit^14^ yields 13 nm periodicity with LFO layer thickness of around 8.5 nm and LSMO layer thickness of 4.5 nm per bilayer. In addition, the simulation shows two distinct Kiessig fringes between the 0th and 1st order superlattice peaks. The bilayer is fitted to consist of 37 d_101_ layers of LFO and 20 d_101_ layers of LSMO. Rocking curves recorded of both the substrate and superlattice peaks shown in the inset of Fig. 1c, reveal a FWHM of 85 arcseconds for DSO and an average of 95 arcseconds for the superlattice peaks, showing well-ordered growth.

Given that DSO imposes anisotropic strain as illustrated in Fig. 1b, it is important to probe how this anisotropy impacts the thin film in the plane. High tensile strain $\:{\left[010\right]}_{\mathrm{o}}$ axis is denoted as Q_x_, in which $\:{\left(424\right)}_{o}^{+}\:$and $\:(5\overline{1}2)_{o}^{-}$ diffraction peaks were chosen for reciprocal space maps, where the superscripts ‘+’ and ‘-‘ represent, respectively, grazing exit and grazing incidence geometry. Further, the compressive $\:[\overline{1}01]_{\mathrm{o}}$ axis is denoted as Q_y_, and the $\:{\left(600\right)}_{o}^{+}$ diffraction peak was investigated. The reciprocal space maps (Fig. 1d-f) show vertically aligned superlattice peaks that are coherent with the observation from the 2theta-omega scan, confirming highly ordered crystal planes in Q_z_ ([101]_o_ out of plane) axis. Since the $\:{\left(600\right)}_{\mathrm{o}}^{+}$ reciprocal space map in Fig. 1e shows vertically aligned superlattice peaks with the substrate peak in the Q_y_ direction, the films are strained in the $\:[\overline{1}01]_{\mathrm{o}}$ axis. However, in the Q_x_ $\:{\left[010\right]}_{\mathrm{o}}\:$direction showing the$\:{\:\left(424\right)}_{\mathrm{o}}^{+}$and $\:(5\overline{1}2)_{o}^{-}$diffraction peaks (Fig. 1d and f), the superlattice peaks do not align with respect to the substrate. Hence, relaxation of the epilayers is present in the Q_x_ $\:{\left[010\right]}_{\mathrm{o}}\:$direction.

The superlattice peaks for Q_x_ $\:{\left[010\right]}_{\mathrm{o}}\:$in Fig. 1d are broad with horizontal width of 0.0158 r.l.u (relative lattice unit) for the 0th peak versus 0.0083 r.l.u for the substrate peak. This broadening of superlattice peaks might arise from mosaicity or structural domains in the epitaxial thin films. Mosaicity in thin films are usually due to disorder of the epilayers which should be visible from rocking curves resulting in large FWHM. No observed broadening in the rocking curves in superlattice peaks in inset of Fig. 1c suggest that the broad superlattice peaks from the reciprocal space maps are due to structural domains.

Average lattice parameters from the reciprocal space maps in Fig. 1d-f were calculated to be a = 0.554 nm, b = 0.558 nm and c = 0.783 nm assuming a fully orthorhombic system even though LFO grown with large lattice mismatch is simulated to have monoclinic or triclinic structure^5,15^. The estimated superlattice parameters are similar to LFO bulk values (a = 0.558 nm, b = 0.557 nm and c = 0.783 nm^16^, which suggests that relaxation is governed by the LFO layers and LSMO is strained to LFO. The exact structure of each layer is hard to determine as the superlattice peak averages the two layers in X-ray diffraction.

**Fig. 2:**
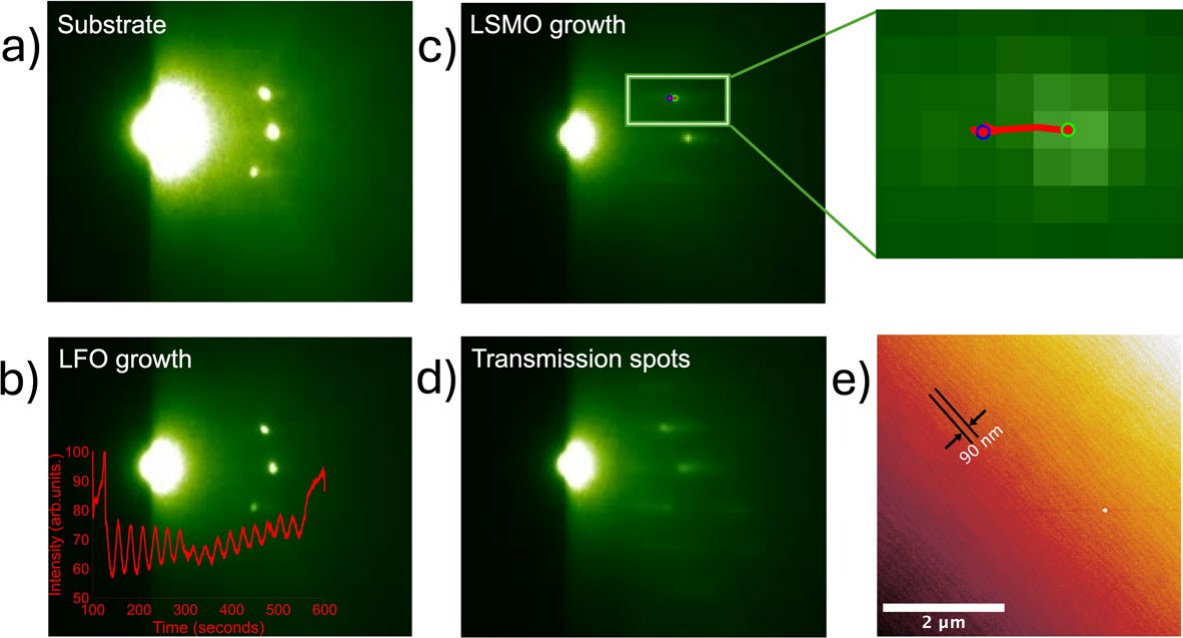
(**a**) RHEED pattern of substrate. (**b**) RHEED pattern with overlay showing the intensity oscillation during LFO layer growth. (**c**) RHEED pattern from initiation of LSMO growth with a horizontal displacement of the side spot on top. Higher magnification inset shows the movement trajectory in a red line with green circle as start and blue circle as the end positions. (**d**) Transition RHEED pattern of LSMO growth. (**e**) AFM image of the superstructure topography with size of step-terraces shown by arrows, with the step width following the substrate.

The RHEED diffraction patterns in Fig. 2a-d show several changes during the deposition process. The substrate had a semi-circle pattern typical of a single crystal with atomically smooth surfaces (Fig. 2a), the pattern is slightly asymmetric due to alignment. The diffraction pattern remained unchanged in position with oscillating intensities during the growth of the initial LFO layer (Fig. 2b). The recorded RHEED intensity shows 16 oscillations throughout the first layer, while the simulation from X-ray diffraction in Fig. 1c yields in average 37 d_101_ layers. Thus, each recorded intensity oscillation corresponds to approximately two d_101_ LFO layers deposited during growth.

At the onset of LSMO growth (Fig. 2c), a horizontal displacement in pixel position of the top spot was observed within the span of seconds. In the higher magnification inset in Fig. 2c, the RHEED spot has been moved from right (green circle) to left (blue circle) showing a rapid change in lattice spacing, suggesting relaxation to occur. However, no observed change in distance between the diffraction spots, shows no in-plane lattice spacing changes. The continuous transition in diffraction pattern from a purely semi-circle to a mixture of transmission and 2D type indicates a change in growth mode from 2D layer-by-layer to partial 3D island growth.

The AFM topography image of the superlattice in Fig. 2e shows relatively smooth surface with mean roughness of 0.28 nm and clear step-and-terraces mimicking the substrate (Figure S1 in Supplementary Information). The AFM image indicates that the growth has not transitioned to large islands growth, but probably smaller islands with 2–3 layers were grown at once resulting in the transmission RHEED pattern. A mixed surface termination of the DSO substrate after preparation as reported by Biegalski et al. might contribute to this growth behaviour^17,18^. The mixed termination is transferred from the substrate to subsequent growth surfaces without no annealing step between each layer growth, impacting the highly surface sensitive LSMO growth and causing islands to form. As the islands coalesce, there is an increased likelihood of strain relaxation in the unfavourable energy [010]_o_ axis. This is coherent with the reciprocal space maps in Fig. 1d-f showing strain relaxation along the tensile strain Q_x_ $\:{\left[010\right]}_{\mathrm{o}}\:$axis. The structural analysis by XRD in Fig. 1 lacks specificity on individual layer level due to averaging nature of the in-house XRD instrument. However, the RHEED recording shows a change in diffraction pattern during the first LSMO layer growth, which call for in-depth localised analysis of the structure.

**Fig. 3:**
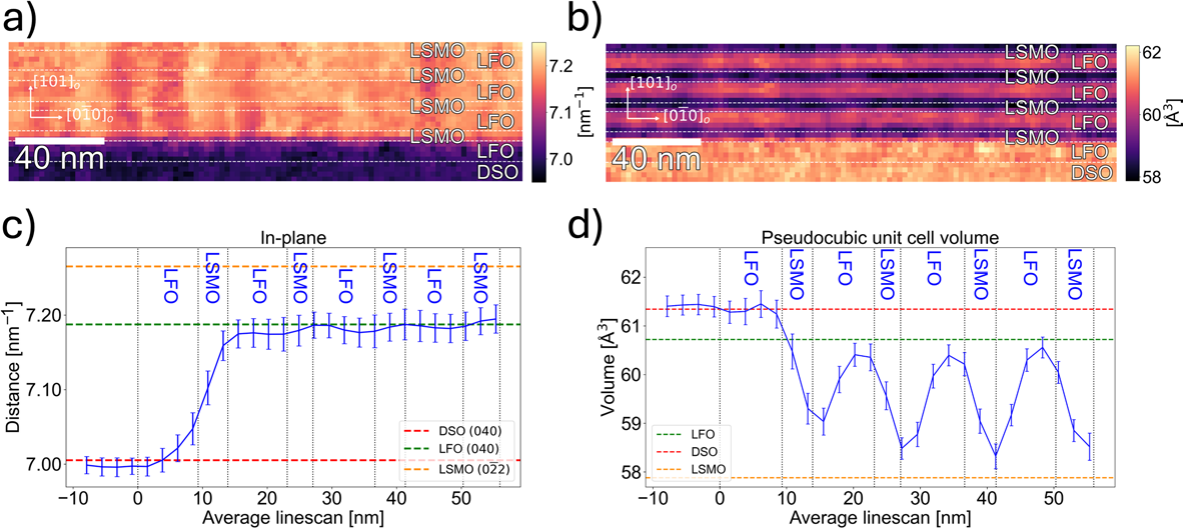
**(a**) Heat map of Friedel pair distance between $\:[040]_{\mathrm{o}}$ and $\:[0\overline{4}0]_{\mathrm{o}}$ Friedel pair, probing in-plane strain in the [010]_o_ axis of the (LSMO/LFO)_4_ superlattice. (**b**) Heat map of pseudocubic unit cell volume based on in- and out-of-plane distances. (**c**) In-plane distances for $\:[040]_{\mathrm{o}}$ and $\:[0\overline{4}0]_{\mathrm{o}}$ Friedel pair with guidelines for DSO, LFO and LSMO lattice parameters, showing the distance converges towards LFO bulk value^16,19,20^. (**d**) Pseudocubic unit cell volume calculated from lattice parameters obtained from the strain analysis. Guidelines are bulk unit cell volumes for DSO, LFO and LSMO^16,19,20^.

Segmented scanning precession electron diffraction (S-SPED) with the beam parallel to the $\:[\overline{1}01]_{\mathrm{o}}$ zone axis (low strain axis) yielding in- and out-of-plane information was employed to analyse local structural strain^12^. From the S-SPED analysis shown in Fig. 3, a thickness of 56 nm was measured for the superstructure which is in line with XRD data, with each bilayer to be around 13–14 nm. The in-plane strain analysis done by measuring the Friedel pair distance (lattice parameter) between $\:[040]_{\mathrm{o}}$ and $\:[0\overline{4}0]_{\mathrm{o}}$ Friedel pair (Figure S2-SI) resulted in the heat map in Fig. 3a. The substrate and first LFO layer have approximately the same Friedel pair distance (Fig. 3c), showing that the first LFO layer was coherently strained to the substrate. A significant increase in the Friedel pair distance in the first LSMO layer was observed and can be interpreted as in-plane lattice parameter variation, showing the starting point of relaxation. This observation is coherent with the RHEED data in Fig. 2 and supports that relaxation occurred first when LSMO was deposited. The following LFO and LSMO layers have almost constant in-plane distances with values around the bulk LFO lattice parameters^16^ as seen in Fig. 3c. Hence no further relaxation occurs, suggesting that the growth front after the first bilayer wasstrained to the LFO layer.

Heat map of the unit cell volume generated by using lattice parameters measured for each axis (Figure S3-SI) is shown in Fig. 3b. The unit cell volume oscillates between the layers. Electron energy loss spectroscopy (EELS) in Figure S4-SI does not show chemical changes within the layers, thus the volume variation is of structural origin. The variation in unit cell volume across the superlattice is shown in Fig. 3d, in which the first LFO layer has the same unit cell volume as the substrate, with a transition to a lower unit cell volume in the first LSMO layer in conjunction to the observed decrease in in-plane [010]_o_ lattice parameter. The subsequent layers show oscillating unit cell volumes that approaches the respective bulk values due to strain relaxation with offset towards LFO bulk value. This suggests that LSMO did not fully relax to its bulk state but is strained towards the LFO lattice parameters.

Strain analysis performed in the perpendicular zone axis relative to data in Fig. 3, probing the $\:[\overline{1}01]_{\mathrm{o}}$ zone axis with compressive strain, showed no relaxation for the in-plane parameters (Figure S5-SI) which is consistent with the XRD data in Fig. 1e. Thus, we can conclude the thin film underwent a partial relaxation of the high-strain axis during the first LSMO layer, and subsequent layers were strained to the second LFO layer.

### Structural domains

**Fig. 4:**
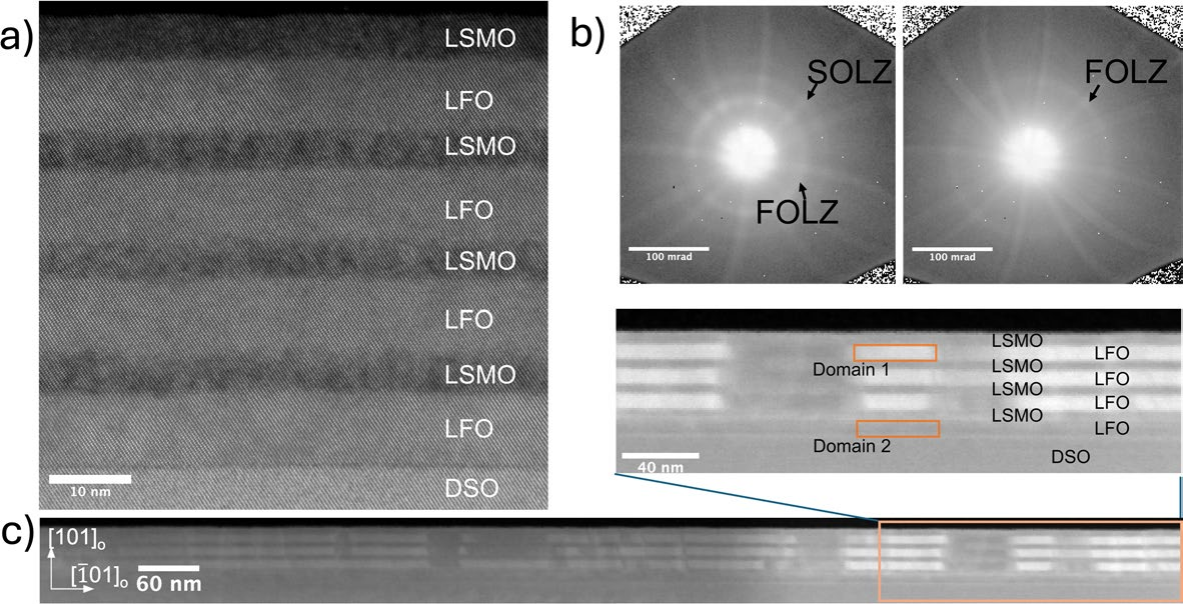
(**a**) STEM-HAADF of the (LSMO/LFO)_4_ superlattice. (**b**) STEM-HOLZ scattering rings from domain 1 on the left and domain 2 on the right. (**c**) Structural domain in 1 μm field of view and zoomed in to 200 nm. Domain 1 is highlighted by selected FOLZ ring in domain 1 as default intensity. Domain 2 has dark appearance due to absence of selected domain 1 FOLZ ring. In addition, a thickness contrast within the lamella is present from FIB fabrication giving a brighter contrast on the right hand side in the image.

The crystal structure of the superlattice was further studied by scanning transmission electron microscopy – high angle annular dark field (STEM-HAADF) as shown in Fig. 4a. Continuous lattice planes with clear interfaces between the layers were observed with no apparent dislocations or other structural defects in the strained [010]_o_ zone axis. In addition, EELS in Figure S4-SI shows no significant intermixing of B-site cations at the interfaces. The STEM data in Fig. 4a shows highly crystalline lattices in the two spatial dimensions perpendicular to the electron beam, but information of the crystal structure parallel to the electron beam is limited. STEM-HOLZ technique that overcomes such spatial limitation was employed to investigate the three-dimensional crystal structure. STEM-HOLZ has previously been used in this way to analyse A-cation ordering and oxygen octahedral tilting in complex oxide thin films^15,21^.

Scanning transmission electron microscopy - higher order Laue zone diffraction, STEM-HOLZ results in Fig. 4b-c show contrasts due to two groups of HOLZ scattering rings defined as domains 1 and 2. These diffraction rings correspond to reflections of the lattice plane along the beam and represents the lattice stacking order. The difference in contrast in HOLZ observed can be attributed to the domain formation minimising the overall strain energy, avoiding structural defects such as misfit dislocations. Domain 1 consists of two rings that are the first (FOLZ) and second (SOLZ) order Laue zone scattering, whereas domain 2 only has one single outer ring (Fig. 4b). To distinguish these two domains, the FOLZ ring in domain 1 was selected as the default contrast intensity. The presence of the two domains is seen by scanning across the lamella of the superstructure in 1 μm field of view and zoomed in to 200 nm as presented in Fig. 4c. The substrate shows homogeneous contrast as expected from a single crystal. The first bilayer shows similar homogeneity which is consistent with a strained LFO and partially relaxed LSMO. A horizontal contrast difference is visible in the LFO layers starting from the second bilayer, confirming the presence of two different types of unit cell ordering (domains) within each layer of LFO. This suggests that the strain relaxation occurring in the first LSMO layer introduces possible structural degeneracy in LFO. The domain contrast patterns have defined vertical boundaries as observed in Fig. 4c. The domain width is in the range from 40 to 100 nm. The domain width range is in good agreement with the step width of the DSO substrates (Figure S1-SI). This observation suggests that the domains nucleate from step edges during growth.

**Fig. 5:**
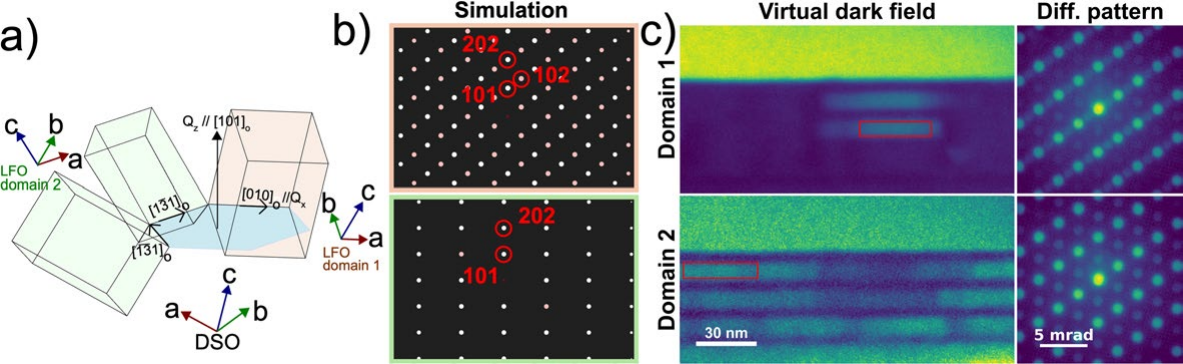
**(a**) Schematic representation of the two possible LFO domain orientations with growth axes annotated with respect to DSO substrate. Domain 2 has a structural degeneracy. (**b**) Diffraction simulation of the two domains in LFO by Recipro^22^. Top and bottom represent, respectively, domain 1 and 2. (**c**) Virtual dark field image reconstructed from S-SPED data by selecting super reflection exclusively to the respective domain, highlighting the domain location throughout the superlattice.

LSMO/LFO heterostructures on (111)SrTiO_3_ substrate reported by Nord et al. show LFO with six-fold degenerate growth orientation resulting in structural twin domains in LFO depending on growth orientation^15^. Similar diffraction rings and domain contrasts as in Fig. 4c were reported, interpreted as unit cell doubling from the structural twin domains. This suggests that the HOLZ scattering contrast observed in our LMSO/LFO/DSO system also represents structural domains with a doubling of the unit cell periodicity in the LFO layers. The area in the lamella with dimmed contrast due to larger thickness, makes it difficult to quantify domain width in that area. The schematic in Fig. 5a illustrates three possible structural orientations of LFO and represents the two types of domains shown in STEM-HOLZ data in Fig. 4. The bulk LFO a_o_ axis is illustrated to be parallel to the DSO (101)_o_ growth surface as seen in Fig. 1b.

The virtual dark field images acquired by S-SPED with simulated diffraction patterns as presented in Fig. 5b-c were used to understand the structural symmetries of each domain. Figure 5b shows simulated diffraction patterns of LFO on DSO (101)_o_ growth surface. The top diffraction pattern is defined as domain 1 with LFO a_o_ axis parallel to [010]_o_ DSO axis. The periodicity doubling in the diffraction pattern along the [001]_o_ direction, showing the unit cell doubling as discussed above^15^. The bottom diffraction pattern is defined as domain 2 with LFO a_o_ axis parallel to either $\:[1\overline{3}1]_{\mathrm{o}}$ or $\:[13\overline{1}]_{\mathrm{o}}$. These two are structurally degenerated and yields indistinguishable diffraction patterns. In addition, each structural domain can have a 90-degree rotation along the c axis resulting in structural twins for each geometry that are indifferent in terms of diffraction. Therefore, it is possible to have b_o_ parallel to these growth axes.

Virtual dark field (VDF) images in Fig. 5c highlight the domain locations within the lamella by applying virtual apertures. The contrasts are provided by selecting super reflections that are exclusive for each domain configuration. In domain 1, diffraction points from a periodic doubling in the [001]_o_ direction are chosen, resulting in bright regions with the rest of the lamella being dark. Conversely, domain 2 is highlighted by choosing $\:[\overline{1}0\overline{1}]_{\mathrm{o}}$ diffraction point. The observed domains are in agreement with STEM-HOLZ data (Fig. 4c) and shows clear presence of two distinctive structural domains in the superlattice. The domains in the LFO layers were translated vertically through the superstructure as also observed in STEM-HOLZ data in Fig. 4b. Zhu et al. observed similar type of structural domain walls in thin LFO film grown on (100) SrTiO_3_, which is in agreement with the results by Nord et al.^23,15^ Further, Zhu et al. showed that strained LFO grown on (001) DyScO_3_ lifts the structural degeneracy and results in structural monodomain. The first LFO layer closest to substrate in the present superlattice is of the same structural monodomain nature, however, polydomain was observed in the superlattice after relaxation occurred in LSMO layer. The LSMO layers did not show any structural domain contrast in VDF reconstruction (Figure S6-SI). This type of layer selective domain that translates across layers with vertical domain walls (Figs. 4c and 5c) has not been reported to the authors knowledge.

From the TEM data in Figs. 4 and 5, strain and structural domain formation are clearly connected. The first LFO layer with fully strained configuration did not have any structural domains. Strain relaxation was observed in the first LSMO layer as seen from RHEED in Fig. 2c and strain analysis from S-SPED in Fig. 3a. This establishes a basis for partial relaxation in subsequent LFO layers and the possibility for domain growth with nucleation from step edges of the substrate.

### Magnetic properties

**Fig. 6:**
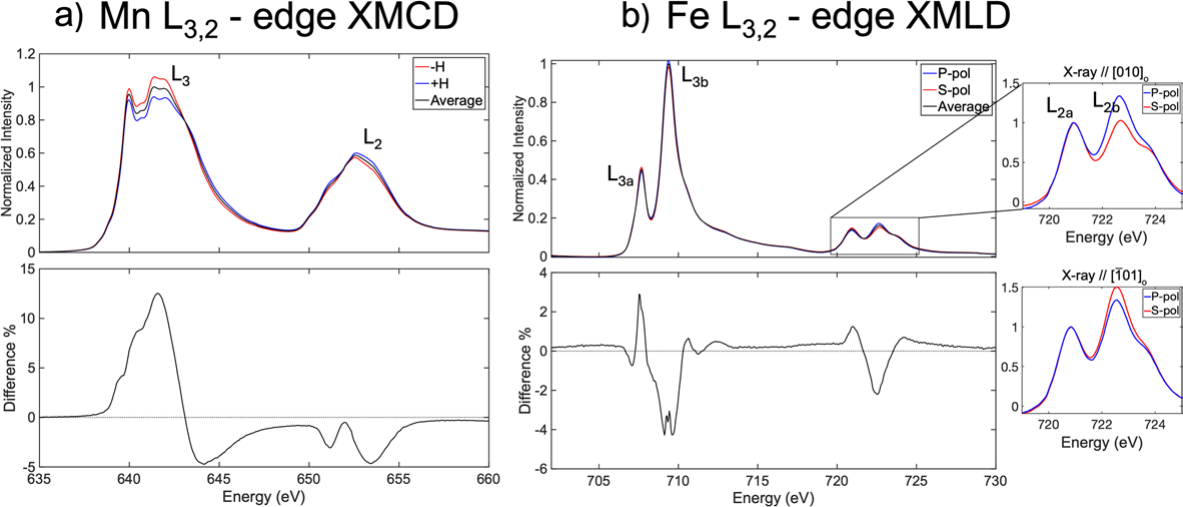
(**a**) Mn L_3,2_ - edge X-ray absorption, with two polarizations in red and blue, with the average XAS in black on top and the resulting difference spectrum between the two polarizations at the bottom, (**b**) Fe L_3,2_ - edge X-ray absorption spectrum with linear polarisation along [010]_o_ (p-polarization in blue, and s-polarization in red) and resulting difference spectrum. The insets to the right show renormalised L_2_ edge with X-ray along [010]_o_ hard axis (top) and $\:[\overline{1}01]_{\mathrm{o}}$] easy axis(bottom). All data is taken at 80 K in total electron yield.

LSMO and LFO have magnetism closely tied to the structural arrangement, hence it is interesting to investigate how the structural domains and relaxation impact the magnetic order of each material. X-ray absorption spectra (XAS) in total electron yield mode (TEY) on top, and magnetic dichroism spectra based on the absorption data at the bottom for LSMO and LFO are provided in Fig. 6a-b. The X-ray magnetic circular dichroism (XMCD) asymmetry at the bottom part of Fig. 6a shows a prototypical in-plane LSMO magnetic signature as previously reported, but with significantly lower signal magnitude^10^. In addition, the L3 peak shape in the XAS indicate a reduction of Mn ions on the surface due to surface sensitivity of the TEY measurement^24^. This reduction might be the reason for the reduced magnetisation in LSMO observed in the XMCD. The complimentary luminescence yield spectra (Figure S7 - SI) show correct stoichiometry with 2/3 to 1/3 ratio for Mn^3+^/^4+^ for LSMO in the rest of the superlattice with XMCD asymmetry around 15%, which s more as expected for 4 nm thick films of LSMO. The X-ray magnetic linear dichroism (XMLD) spectrum acquired at grazing incidence ($\:{\uptheta\:}$=35°) along the LFO hard axis [010]_o_ at 80 K is shown in Fig. 6b. The inset to the right of Fig. 6b shows L_2_ edge spectra with X-ray along [010]_o_ hard axis (top) and $\:[\overline{1}01]_{\mathrm{o}}$ easy axis (bottom) by 90° azimuth rotation. The L_2_ edge spectra was renormalised with pre-edge to zero and L_2a_ edge to 1 for data consistency. The L_2_ edge spectrum along [010]_o_ hard axis shows equal L_2a_ and L_2b_ peak intensites with s-polarisation and higher peak intensity in L_2b_ versus L_2a_ in p-polarisation. This result is identical to XMLD of (111)_c_-LFO/STO by Hallsteinsen et al.^25^ and (111)_pc_-LFO/NGO by Kjærnes et al.^5^. reporting an AF polydomain structure. Additionally, the L_2_ edge spectrum with 90° azimuth rotation (X-ray along $\:[\overline{1}01]_{\mathrm{o}}$ easy axis at the bottom inset) results in higher L_2b_ intensity with s-polarisation while p-polarisation does not change. This observed change in L_2b_ intensity of s-polarisation with azimuth dependency indicates that the AF axes are predominantly in-plane, i.e., parallel to the sample surface. The higher s-polarisation intensity at L_2b_ peak when X-ray is along $\:[\overline{1}01]_{\mathrm{o}}$ easy axis can be interpreted as a higher domain population with AF axis projected in-plane along [010]_o_ (parallel to the s-polarisation). This result in junction with report by Kjærnes et al. indicates that LFO have concurrent structural and antiferromagnetic domains. Hence our finding with partially relaxed LFO on DSO(101)_o_ shows structural and AF polydomain similar to the case of (111)_pc_-LFO/NGO by Kjærnes et al., meanwhile Kjærnes et al. report structural and AF monodomain for fully strained LFO on DSO(101)_o_^5^.

### Outlook

The dichroism results from Fig. 6 and previous reports^5,25^ show that the structural and antiferromagnetic domains have a symbiotic relationship in LFO/LSMO superstructures, as tensile strained LFO can be either monodomain or polydomain depending on the structural strain state of the film to substrate. In-plane uniaxial AF monodomain is achieved when LFO layers are fully strained to (101)_o_-DSO and polydomain is formed when LFO is in a structural degenerate state on (111)_c_-STO and (111)_pc_-NGO substrates. Thus, by tuning the strain state layer by layer, one can switch between a uniaxial monodomain configuration and a polydomain state. This deterministic AF domain control via strain could be a powerful tool which can be utilized to “write” the antiferromagnetic order during growth.

The structural domains formed under anisotropic strain may also have implications for AF domain wall engineering. Previous work has shown that strain can strongly influence spin textures at domain walls (e.g., NiO on mismatched substrates^26^. By analogy, it is plausible that the domain boundaries in our superstructures affect local spin configurations which could serve as templates for engineered domain-wall behavior. Such walls, if present and being controllable, could act as channels for spin transport or be utilized in devices based on resistance changes at AF boundaries.

## Conclusion

This study shows the presence of anisotropic strain induced structural relaxation and domain formation in complex LSMO/LFO superlattices on (101) oriented DSO. Selective relaxation occurred along the in-plane tensile [010]ₒ axis, while the perpendicular in-plane compressive $\:[\overline{1}01]_{\mathrm{o}}$ axis remained fully strained. As a result, structural domains emerge within the LFO layers, starting from the second bilayer and continuing vertically throughout the film. These domains provide an energetically favorable relaxation pathway without the formation of structural defects, and their nucleation originate with step edges on the substrate surface. Strain analysis and diffraction studies show that the LSMO layers adapt to the relaxed LaFeO_3_ structure, being strained to the LaFeO_3_. Magnetic characterization revealed antiferromagnetic polydomain in LaFeO_3_ with degenerate structural domains. These findings display an interplay between strain state, domain formation, and magnetic ordering. The ability to control strain-driven domain structures opens a pathway toward deterministic manipulation of antiferromagnetic configurations via epitaxial design. This insight paves the way for exploiting anisotropic strain as a functional tool in AF domain engineering and next-generation spintronic devices.

## Experimental section

### Sample growth, X-ray diffraction and AFM

The (La_0.7_Sr_0.3_MnO_3_/LaFeO_3_)_4_ superlattice was deposited by pulsed laser deposition (PLD) (TSST, Twente, NL) on (101)_o_-DyScO_3_ substrate (SurfaceNet GMBH, Germany). The substrate was pretreated with buffered HF etching and subsequent annealing at 1050 °C for 1 h in oxygen similar to previous reports^27^. The substrate temperature during growth was set to 580 °C with a ramp of 15 K/min for both heating and cooling. The O_2_ pressure during heating and growth was 200 mTorr and 75 Torr for in-situ annealing for 1 h. The treated substrate showed a step-and-terrace surface finish with step width range from 40 to 100 nm. A KrF excimer laser (Coherent, US) ((λ = 248 nm) was used to ablate stoichiometric LSMO, LFO targets with a substrate-target distance of 50 mm at 2 Hz. In-situ reflective high energy electron diffraction (RHEED) (STAIB Instruments, Germany) was recorded during the growth process. Atomic force microscopy (AFM) (Bruker, Dimension Icon, US) was used for recording topographical data. The crystalline structure was investigated using four-circle, high resolution X-ray diffractometer (XRD, Bruker D9 Discover, US), with monochromatic Cu Kα1 radiation and 0.2 mm detector slits. Reciprocal space maps (RSM) were collected with position sensitive detector with 0.002° steps in steps in ω. The RSM data were collected from asymmetric reflections $\:{\left(424\right)}_{o}^{+}$, $\:{\left(600\right)}_{o}^{+}$ and $\:(5\overline{1}2)_{o}^{-}$ as well as the symmetric (202)_o_ reflection, with grazing exit (+) and incidence (-) geometries to account for limited signal intensity.

### TEM and lamella Preparation

The TEM lamella was fabricated with Thermo Fisher Helios G4UX focused ion beam (FIB) (US) with standard lamella preparation method. A carbon-based protection layer was deposited to protect the thin film from Ga ion contamination, which also served as alignment tool for S-SPED data analysis.

Segmented scanning precession electron diffraction (S-SPED) datasets were acquired on a JEOL JEM-2100 F (Japan), in the nanobeam diffraction mode with a convergence angle of 1.2 mrad, 1 degree precession angle and 100 Hz frequency. STEM-HOLZ and atomic resolution data were acquired on a JEOL ARM-200CF. Both instruments were equipped with a MerlinEM direct electron detector and were operated at 200 kV. Post-acquisition data processing was performed using the open-source Python libraries HyperSpy^28^ and pyxem^29^.

The S-S(P)ED data were acquired with a nominal probe size of 1.39 nm, which increased with the application of precession, and a step size of 0.92 nm. Virtual dark field (VDF) images were reconstructed from the sliced segments of the S-SPED scan and used to manually approximate the probe shifts based on feature movement with the assistance of the amorphous carbon layer.

The strain analysis was carried out by choosing a Friedel pair (*hkl*) and $\:(\overline{\mathrm{hkl}})$ for each lattice spacing of interest. The pixel size calibration of the detector was constant throughout the region of interest. For in-plane strain analysis, the (040)_o_ and $\:(0\overline{4}0)$ reflections of zone axis $\:[\overline{1}01]_{\mathrm{o}}$ were used, and the (202)_o_ and $\:(\overline{2}0\overline{2})_\mathrm{o}$ reflections were used out-of-plane analysis.

### X-ray absorption

X-ray circular and linear dichroism data were acquired at beamline 4.0.2 at Advanced Light Source, LBNL, US in total electron yield and luminescence yield mode providing both surface magnetic sensitivity and bulk chemical composition. The experiments were done in a vector magnet chamber at 80 K cooled by liquid nitrogen with X-rays at grazing incidence angle of 35° with respect to sample surface. Total electron yield measurements for Mn were carried out with ± 0.3T switching magnetic field parallel to sample surface along the beam direction and right circular polarisation. Luminescence yield measurements for Mn were carried out with ± switching circular polarisation^30,31^. Total electron yield measurements for Fe were carried out with 0.3T magnetic field parallel to sample surface along the beam direction with ± switching linear polarisation.

## Supplementary Information

Below is the link to the electronic supplementary material.

Supplementary Material 1

## Data Availability

The datasets used and/or analysed during the current study available from the corresponding author on reasonable request.
