# Supplementary material for: Structural relaxation and domain formation in anisotropically strained La0.7Sr0.3MnO3/LaFeO3 superlattices on DyScO3(101)
Source: Sci Rep. 2026 Jan 13;16:5123. doi: 10.1038/s41598-026-35436-2 (PMC12877136; doi:10.1038/s41598-026-35436-2)
Supplement: Supplementary file 1 — Supplementary Material 1 [file 41598_2026_35436_MOESM1_ESM.pdf]

Supplementary Information

Anisotropic Strain Engineering in $La_{0.7}Sr_{0.3}MnO_3/LaFeO_3$ Superlattice: Structural Relaxation and Domain Formation

*Yu Liu, Thea Marie Dale, Emma van der Minne, Susanne Boucher, Romar Avila, Christoph Klewe, Gertjan Koster, Magnus Nord, Mari-Ann Einarsrud, Ingrid Hallsteinsen**

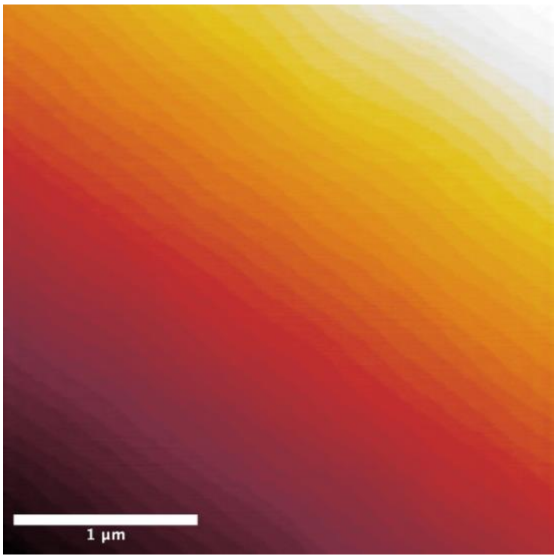

Figure S1) Atomic force microscopy image of the $DyScO_3$ substrate showing clear step-and-terrace topography after surface treatment.

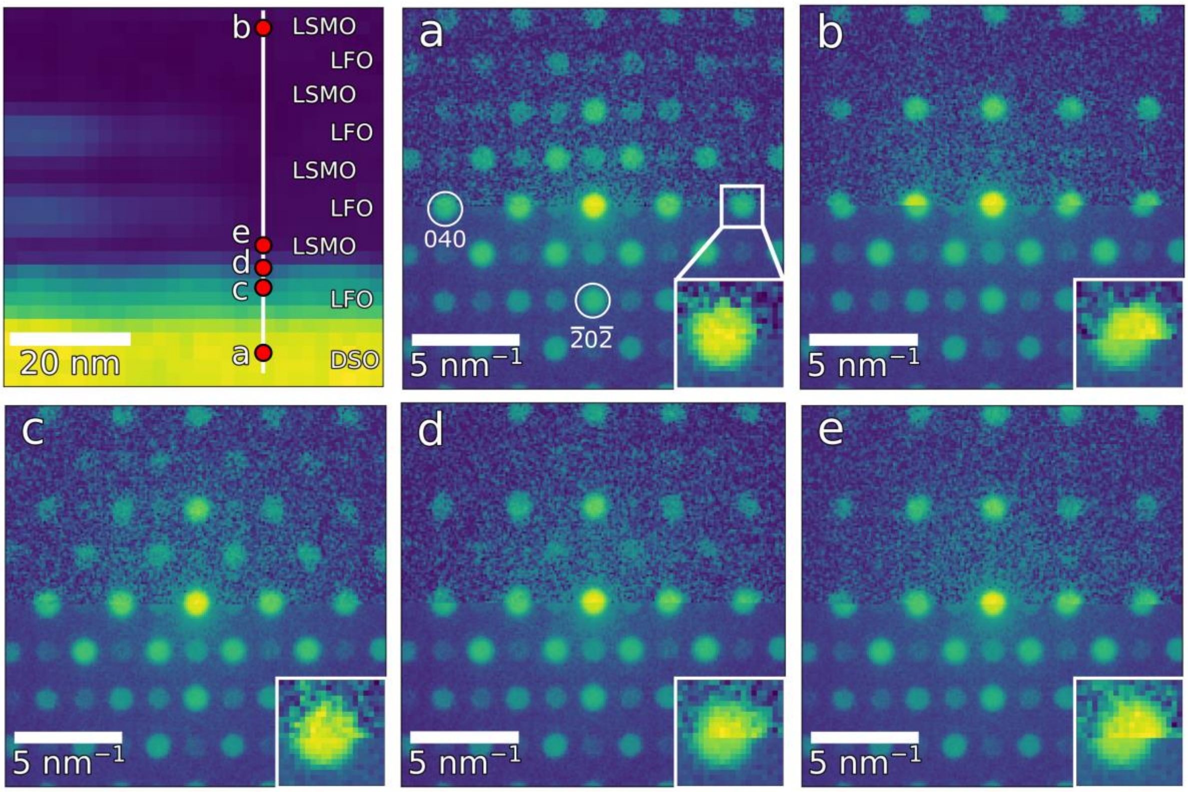

Figure S2) In-plane strain analysis of the [040]//[0$\bar{4}$0] Friedel pair with substrate distance as reference. Line profile with markings showing regions investigated. a) Reference distance of DSO substrate. b) Topmost layer of LSMO (upper part) compared with DSO (lower part) showing a significant displacement (zoom in of [0$\bar{4}$0]) in diffraction point due to relaxation. c) First layer of LFO (upper) compared with DSO (lower), insignificant pixel shift is seen which is in the tolerance margin. d) Beginning of the first LSMO layer (upper) compared with DSO (lower) showing significant shift in position from c), suggests a relaxation has occurred at this layer. e) End of the first LSMO layer (upper) compared with DSO (lower) showing an increased position shift from d) due to extended relaxation. However, the position is compared to b) which suggests the degree (magnitude) of relaxation is stabilised after this point for the remainder of the superlattice.

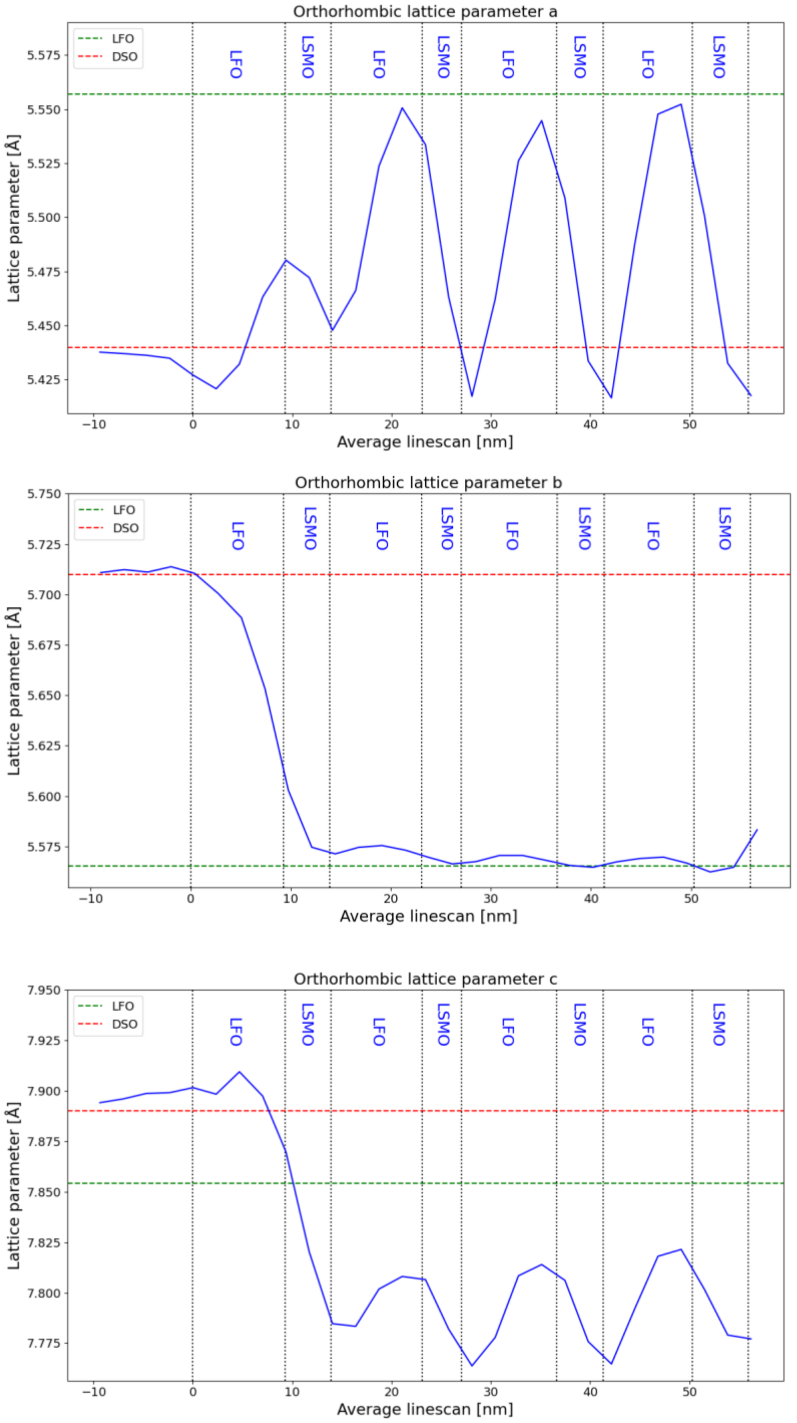

Figure S3) Measured orthorhombic lattice parameter a, b, c from Friedel pair diffraction points [040]//[$0\bar{4}0$] form [$\bar{1}01$] zone axis , [202]//[$\bar{2}0\bar{2}$] and [026]//[$0\bar{2}\bar{6}$] from [$13\bar{1}$] zone axis. The lattice parameter is half of the separation in distance between the diffraction pair.

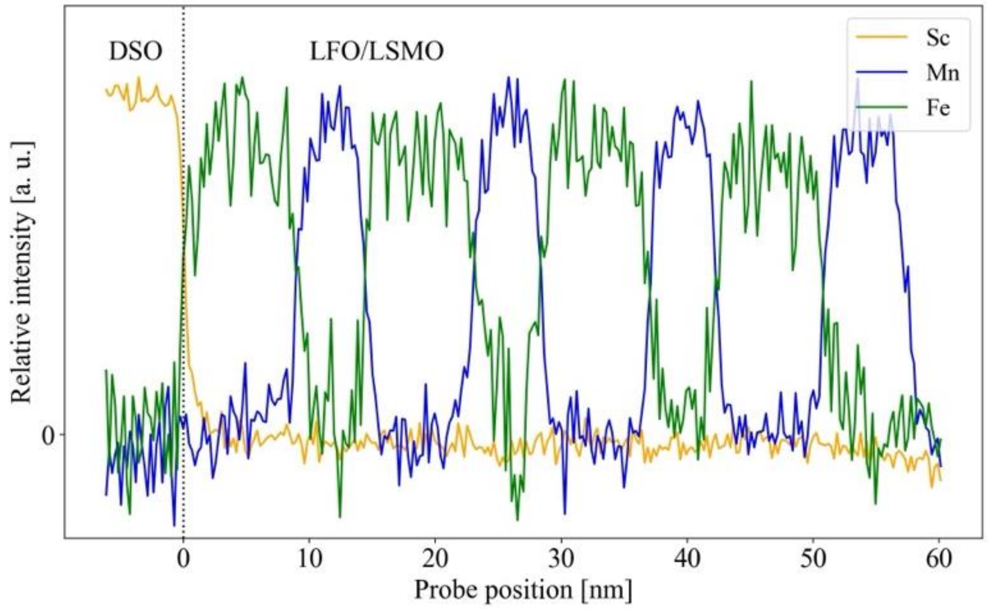

Figure S4) Scanning transmission electron microscopy - Electron energy loss spectroscopy line scan across the superlattice and substrate. Integrated intensities of the B-site cation edges (Sc, Mn and Fe), giving the relative element content.

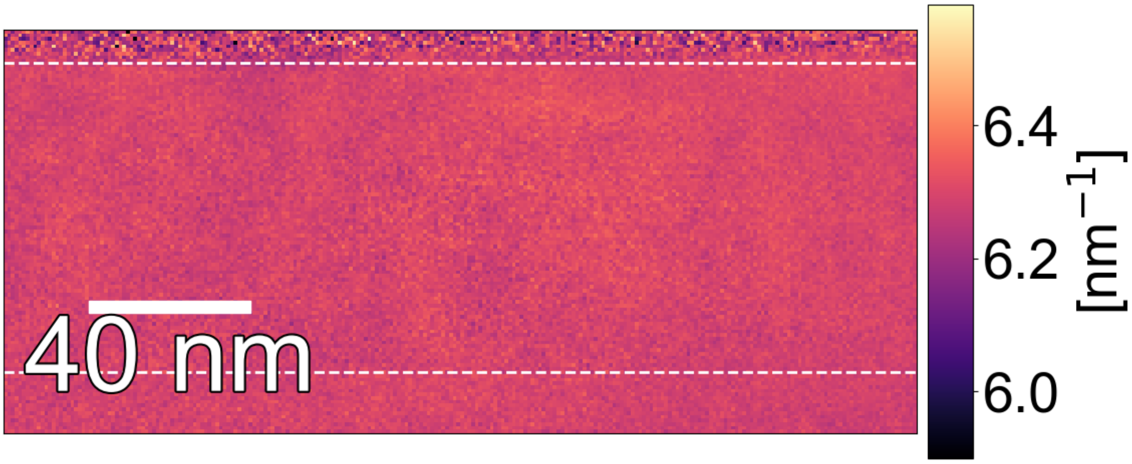

Figure S5) Heat map from Friedel pair $[20\bar{4}]//[\bar{2}0\bar{4}]$, showing the in-plane lattice parameter variation in the $[\bar{1}01]$ zone axis with the dotted lines indicating the superlattice location. The highly uniform heat map suggests that no relaxation is present in this zone axis.

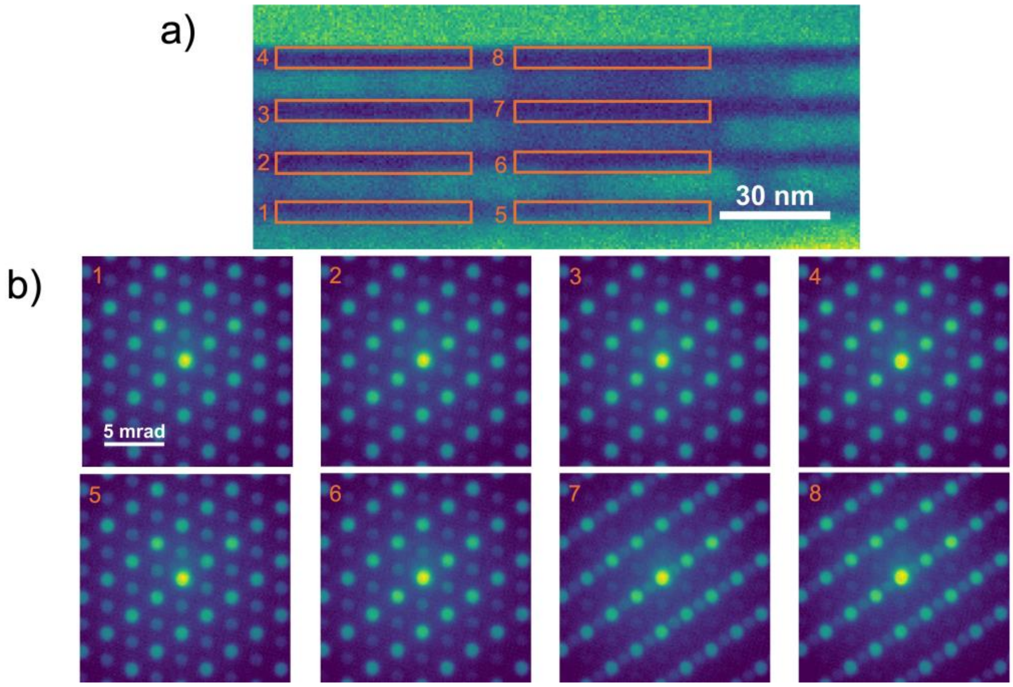

Figure S6) a) Virtual dark field image of the lamella with $(111)_{pc}$ virtual aperture, showing no domain contrasts within the LSMO layers. Multiple regions are selected for manual inspection b) virtual diffraction patterns from regions in VDF image above, showing no real structural changes within the layers. A faint superreflection is observed in 7-8) near LFO domain 1, which might arise from dynamic scattering effects instead of structural changes.

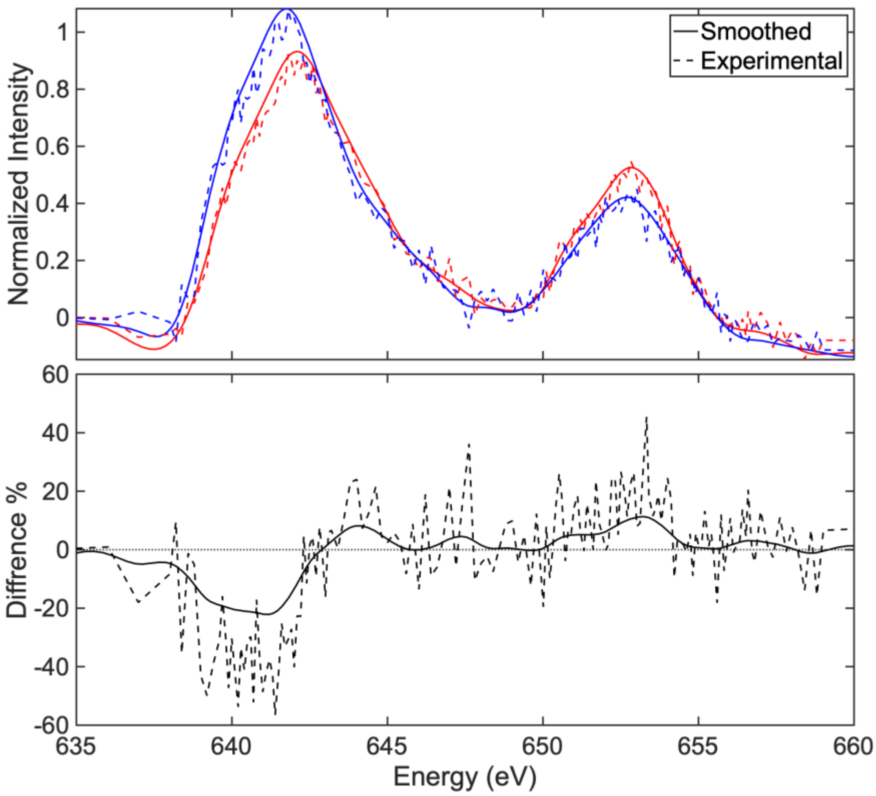

Figure S7) a) X-ray absorption spectra in circular polarisation for Mn energy. The observed peak splitting from reduction of Mn^{3+} in TEY mode is absent, showing bulk LSMO layers retains 2/3 to 1/3 ratio for $Mn^{3+/4+}$. b) Calculated X-ray magnetic circular dichroism (XMCD) showing an asymmetry ratio around 15 % that is in range for LSMO thin films. Smoothed XMCD is shown in blue from Smooth Spline fit function.
